# Quantitative proteome changes in *Arabidopsis thaliana* suspension-cultured cells in response to plant natriuretic peptides

**DOI:** 10.1016/j.dib.2015.06.013

**Published:** 2015-06-30

**Authors:** Ilona Turek, Janet I. Wheeler, Chris Gehring, Helen R. Irving, Claudius Marondedze

**Affiliations:** a Division of Biological and Environmental Science and Engineering, King Abdullah University of Science and Technology, Thuwal, Saudi Arabia; b Drug Discovery Biology, Monash Institute of Pharmaceutical Sciences, Monash University, Melbourne, VIC, Australia; c Cambridge Centre for Proteomics, Department of Biochemistry, University of Cambridge, Cambridge, United Kingdom

**Keywords:** Plant natriuretic peptide, Quantitative proteomics, Peptide hormone signaling, Plant homeostasis, Molecular mimicry, Salt stress, Reactive oxygen species

## Abstract

Proteome changes in the *Arabidopsis thaliana* suspension cells in response to the *A. thaliana* plant natriuretic peptide (PNP), AtPNP-A (At2g18660) were assessed using quantitative proteomics employing tandem mass tag (TMT) labeling and tandem mass spectrometry (LC–MS/MS). In this study, we characterized temporal responses of suspension-cultured cells to 1 nM and 10 pM AtPNP-A at 0, 10 and 30 min post-treatment. Both concentrations we found to yield a distinct differential proteome signature. The data shown in this article are associated with the article “Plant natriuretic peptides induce a specific set of proteins diagnostic for an adaptive response to abiotic stress” by Turek et al. (Front. Plant Sci. 5 (2014) 661) and have been deposited to the ProteomeXchange with identifier PXD001386.

**Specifications table**

**Table:** 

Subject area	Biology
More specific subject area	Plant science, Arabidopsis cell suspension proteome
Type of data	MS data and annotations
How data was acquired	TMT labeled peptides were analyzed using LTQ Orbitrap Velos mass spectrometer (Thermo Fisher Scientific, Germany)
Data format	Analyzed output data
Experimental factors	*A. thaliana* suspension-cultured cells were treated with AtPNP-A or water (mock treatment) and total protein was extracted, digested with trypsin and the peptides were labeled with TMT
Experimental features	The peptides resulting from in-solution tryptic digestion of total proteins from treated cells were labeled with TMT, OFFGEL-fractionated and analyzed using LC–MS/MS
Data source location	Thuwal, Saudi Arabia
Data accessibility	The data available in this article is related to [1] and deposited to the ProteomeXchange with identifier PXD001386 (http://proteomecentral.proteomexchange.org/cgi/GetDataset?ID=PXD001386)

**Value of the data**

• A total of 4641 proteins were identified in response to 1 nM AtPNP-A and 3447 proteins in response to 10 pM AtPNP-A at a false discovery rate (FDR) of 0.7% for protein and 1.6% for peptide.
• 11 proteins are differentially expressed in response to 1 nM AtPNP-A and 15 proteins in response to 10 pM AtPNP-A. These differentially expressed proteins are mainly enriched for functional categories of translation and response to salt, heat and oxidative stress.
• Cellular responses to PNPs are highly concentration-dependent.
• We propose that AtPNP-A, possibly signaling through cGMP, has a key role in oxidation-reduction processes as well as response to salt stress.
• The data are valuable for understanding the molecular mechanism of AtPNP-A action and are key for further exploration of the PNP signaling.

## 1 Experimental design

**Fig. 1:**
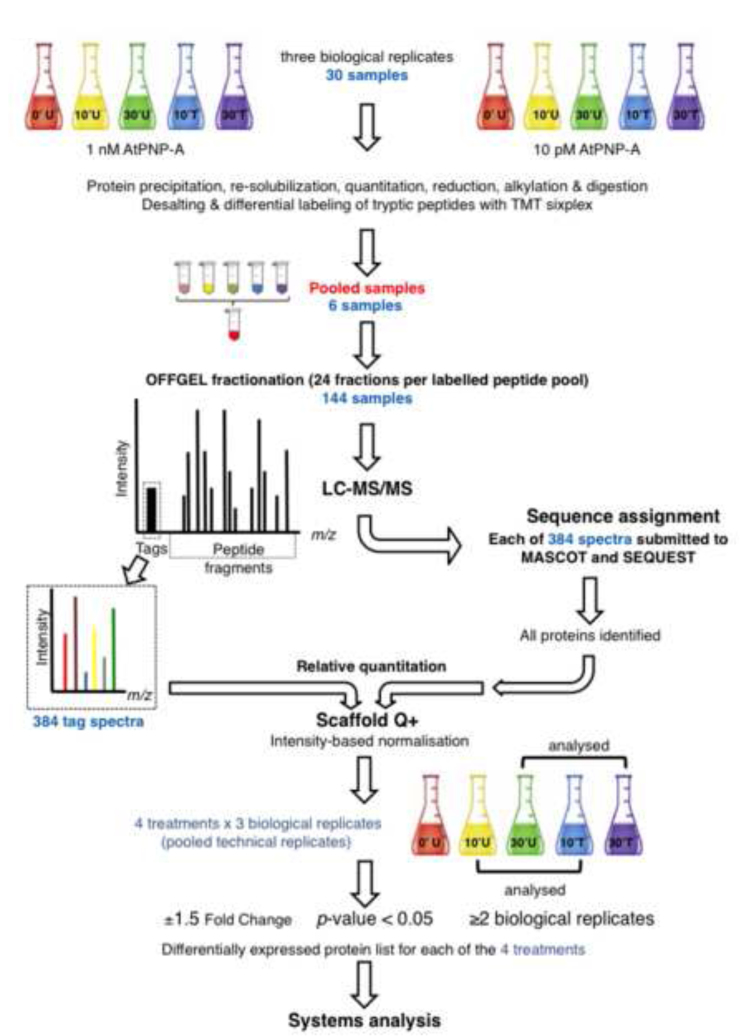
Overview of the experiment. Arabidopsis cell suspension cultures were treated (T) with AtPNP-A (1 nM or 10 pM) or water (mock, U) for 0, 10 or 30 min. Three biological replicates were performed for each, resulting in 30 samples. Protein extraction, quantitation, reduction, alkylation and digestion were performed for each samples followed by desalting before differential labeling of the tryptic peptides with TMT sixplex. AtPNP-A-treated cells collected at 10 and 30 min post-treatment were labeled with *m*/*z* 129 TMT and *m*/*z* 130 TMT while the 0, 10, 30 min mock treated cells were labeled with *m*/*z* 126 TMT, *m*/*z* 127 TMT and *m*/*z* 128 TMT, respectively. Equal amounts of labeled peptides from the corresponding biological replicates were then pooled to create 6 combined samples (3 biological replicates for 1 nM AtPNP-A treatments and 3 biological replicates for 10 pM AtPNP-A treatments). OFFGEL fractionation was performed giving rise to 24 fractions per labeled peptide pool. Each of these samples was analyzed by LC/MS–MS. Each spectrum was analyzed independently by MASCOT and SEQUEST for sequence assignment (Supplementary Tables 1 and 2). Scaffold Q+ was then used to relatively quantify all identified proteins. Differential protein expression was considered significant if the combined data from pooled technical replicates for a given biological replicate was greater or equal to |±1.5| of the related mock treatment, verified by Mann–Whitney test (*p*-value <0.05), in at least two out of three biological replicates (Tables 1 and 2). Differentially expressed proteins were then examined by GO and gene expression analysis.

Quantitative proteomic changes in response to *Arabidopsis thaliana* PNP (AtPNP-A; At2g18660) was performed as outlined in Fig. 1. Total soluble proteins were extracted from 10 to 30 min Arabidopsis cell suspension culture samples either treated with water or with different concentrations (1 nM and 10 pM) of AtPNP-A. A total of three biological replicates of the mock-treated and three biological replicates of cells treated with each concentration of AtPNP-A were used per each time-point considered in this study. We performed a proteomic analysis using LTQ Orbitrap Velos after OFFGEL fractionation of the TMT-labeled tryptic peptides. The acquired mass data identification was performed using MASCOT and SEQUEST search engines and the interpretation was done using Scaffold Q+ software. The proteomics data presented here include the protein and spectrum identification results, gene ontology (GO) functional category and transcriptional profiling results. Here, 4641 proteins were identified in response to 1 nM AtPNP-A and 3447 proteins in response to 10 pM AtPNP-A, at FDR of 0.7% for protein and 1.6% for peptide. 11 unique proteins were differentially expressed in response to 1 nM AtPNP-A while expression of 15 proteins was significantly regulated upon treatment with 10 pM AtPNP-A. The functional categories of the proteins with significantly altered expression in response to AtPNP-A were annotated using TAIR GO search (http://www.arabidopsis.org/tools/bulk/go/index.jsp). The transcriptional profile of these proteins was also analyzed using Genevestigator (https://www.genevestigator.com/gv/plant.jsp) [2].

## 2 Materials and methods

### 2.1 Treatment of Arabidopsis cell suspension culture with AtPNP-A peptide

*A. thaliana* (ecotype Columbia-0) cell suspension culture grown in 100 mL of Gamborg’s B-5 [3] media containing 2,4-dichlorophenoxyacetic acid (0.5 μg mL^−1^) and kinetin (0.05 μg mL^−1^) were treated with a biologically active synthetic peptide (GenScript, Piscataway, NJ, USA) containing the active region of AtPNP-A (amino acid 36–69) [4] at the final concentrations of 1 nM and 10 pM or with equal volumes of water as a negative control. Three biological replicates of each mock- or AtPNP-A-treated cells were collected at 0 min and 10 and 30 min post-treatment. Media were drained and the cells were immediately flash frozen in liquid nitrogen and stored at −140 °C until further use.

### 2.2 Total soluble protein extraction and digestion with trypsin

Approximately 1 g of cells was homogenized for 4 s twice in 10 volumes of ice-cold 10% (w/v) trichloroacetic acid in acetone using a PowerGen 125 grinder (Fisher Scientific, Rockford, IL, USA), vortexed and incubated overnight at −20 °C. Precipitated proteins were pelleted by centrifugation using the Allegra^®^ X-22R centrifuge (Beckman Coulter Corp., Brea, CA, USA) at 3901×*g* for 20 min at 4 °C. The pellet was washed four times with 80% (v/v) ice-cold acetone with vigorous vortexing and subjected to centrifugation at 3901×*g* for 20 min at 4 °C after each wash. Excess acetone was evaporated by air-drying, and proteins were re-suspended in two volumes of urea lysis buffer [7 M urea, 2 M thiourea, phosphatase inhibitor cocktail set II (Calbiochem, Temecula, CA, USA)] with vigorous vortexing for 3 h at room temperature. The samples were cleared by centrifugation at 3901×*g* for 20 min at room temperature and total soluble protein concentration was estimated by Bradford assay [5] using the Quick Start™ Bradford reagent (Bio-Rad, Hercules, CA, USA) and bovine serum albumin as a standard. Approximately 1 mg of total soluble protein extract was digested with trypsin and purified using Sep-Pak Vac tC18 100 mg cartridge (Waters, Milford, MA, USA), as described previously [6], and completely dried in a Speed Vac concentrator (Thermo Scientific, Bremen, Germany).

### 2.3 Peptide labeling using tandem mass tag (TMT)

Dried desalted tryptic peptides were re-suspended in 20% (v/v) acetonitrile and half of the volume was subjected to labeling reaction using TMT sixplex^TM^ isobaric mass tagging kit (Thermo Scientific) performed according to the manufacturer’s instructions. Each biological replicate corresponding to either 1 nM or 10 pM AtPNP-A-treated samples was labeled separately with the respective mock-treated samples and analyzed independently. Tryptic digests were derivatized with sixplex chemical labels: mock-treated cells collected at 0 min with *m*/*z* 126 TMT, mock-treated cells at 10 min post-treatment with *m*/*z* 127 TMT, mock-treated cells at 30 min post-treatment with *m*/*z* 128 TMT, AtPNP-A-treated cells collected at 10 min post-treatment with *m*/*z* 129 TMT and AtPNP-A-treated cells collected at 30 min post-treatment with *m*/*z* 130 TMT. After 1 h incubation, reactions were quenched by 15 min incubation with 8 μL of 5% hydroxylamine. The five labeled samples for each biological replicate in each treatment with either 1 nM or 10 pM AtPNP-A were subsequently combined at equal amounts and stored at −80 °C until further use. The protocol outline is shown in Fig. 1.

### 2.4 Peptide fractionation by OFFGEL fractionator

The pooled TMT-labeled peptides were fractionated using the 3100 OFFGEL fractionator (Agilent Technologies, CA, USA) with a 24-well high-resolution immobilized pH gradient strip. Peptide samples were diluted to a final volume of 1.8 mL with 1.25×peptide OFFGEL stock solution [50% (v/v) glycerol solution, 10% (v/v) OFFGEL buffer pH range 3–10]. Strips were rehydrated, as recommended by the manufacturer, and then 150 μL of sample was pipetted into each well. Electrofocusing was carried out to 64 kV h at 20 °C, allowing a maximum of 4500 V and 50 μA per strip. After focusing, fractions were separately collected and the wells rinsed twice with 200 μL of a solution containing 50% (v/v) acetonitrile and 5% (v/v) formic acid for 15 min each time. Rinsing solution collected from each well was combined into the tube containing its corresponding fraction. Sample fractions were dried using a Speed Vac concentrator unless stated otherwise. OFFGEL fractions were dried, re-suspended in 0.1% (v/v) trifluoroacetic acid and desalted with Sep-Pak Vac tC18 cartridge, as previously described [6]. Ten percent (v/v) of each purified fraction was dried and resuspended in 10 μL of 0.1% (v/v) trifluoroacetic acid for purification using ZipTipC_18_ (P-10) tips (EMD Millipore) according to the manufacturer’s recommendations. Peptides were dried in preparation for LC–MS/MS analysis. The protocol outline is shown in Fig. 1.

### 2.5 Protein identification by LTQ Orbitrap

Dried peptides were re-suspended in a solution containing 5% (v/v) acetonitrile and 0.1% (v/v) formic acid and analyzed by an LTQ Orbitrap Velos™ mass spectrometer (Thermo Scientific) operated as described previously [6]. Data were recorded with the Xcalibur software version 2.1 (Thermo Scientific) and converted from “.raw” to “.mgf” with Proteome Discover version 1.2.0.208 (Thermo Scientific). All spectra were submitted to a local MASCOT (Matrix Science, London, UK) and SEQUEST (Thermo Scientific) servers and searched against *A. thaliana* in the TAIR database (release 10), with a precursor mass tolerance of 10 ppm, a fragment ion mass tolerance of ±0.5 Da, and strict trypsin specificity allowing up to one missed cleavage, carbamidomethyl modification on cysteine residues as fixed modification, and oxidation of methionine residues and phosphorylation of serine, threonine and tyrosine residues as variable modifications. Identified proteins were all evaluated and quantified using Scaffold Q+ software, version 4.0.4 (Proteome Software, Portland, USA) (Fig. 1). Important to note is that proteins were considered positive identifications if they were identified with a minimum of one unique peptide (SEQUEST Xcorr >2 or MASCOT ion score >32 and a peptide probability of 90%) at the protein threshold of 95% (Fig. 1).

### 2.6 Quantification of differentially expressed proteins

Quantification of protein abundance was performed with Scaffold Q+ software. Proteins were normalized based on the assumption that total intensity remained the same for each of the tags used, and protein FDR of 0.7% was used. Expression levels of positively identified proteins from AtPNP-A-treated cells (present in at least one technical replicate) were compared with mock-treated cells collected at the same time-point. Differential expression of a protein was considered significant if the fold change was greater or equal to |±1.5|, verified by Mann–Whitney test (*p*-value <0.05), in at least two out of three biological replicates. The information is integrated in Tables 1 and 2 as well as Supplementary Tables 1 and 2.

### 2.7 Gene ontology (GO) and gene expression analyzes

**Fig. 2:**
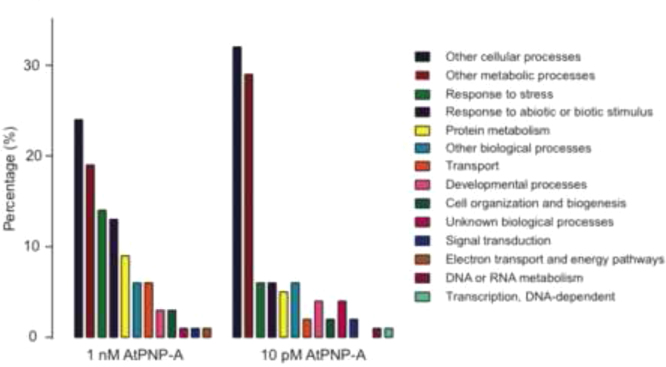
Comparison of GO categories differentially regulated by AtPNP-A at 1 nM and 10 pM concentration.

GO and functional categorization analyzes of proteins significantly differentially expressed were performed using TAIR GO search (http://www.arabidopsis.org/tools/bulk/go/index.jsp; October 2014). This data is represented in Fig. 2 as well as Tables 1 and 2. Transcriptional profiles of proteins that are affected by AtPNP-A were analyzed using Genevestigator (https://www.genevestigator.com/gv/plant.jsp [2]; February 2014).

The mass spectrometry proteomics data was deposited to the ProteomeXchange Consortium [7] via the PRIDE partner repository with the dataset identifier PXD001386 and DOI 10.6019/PXD001386

## Figures and Tables

**Table 1:** Annotation of proteins differentially expressed 10 min and 30 min after treatment of cells with 1 nM AtPNP-A peptide.

**AGI ID**	**Protein annotation**	***p*****-Value**	**Log**_**2**_**fold change**	**GO term (BP)**
**Proteins with expression differentially regulated 10** **min post-treatment**				
AT2G37230.1	Tetratricopeptide repeat (TPR)-like superfamily protein	0.0001	1.10	
AT1G52300.1	Zinc-binding ribosomal protein family protein (RPL37B)	0.0001	1.05	A
AT2G32120.1	Heat-shock protein 70T-2 (HSP70T-2)	0.0001	0.95	
AT5G07470.1	Peptide methionine sulfoxide reductase 3 (PMSR3)	0.0001	0.95	B, C
AT3G58640.1	Mitogen activated protein kinase kinase kinase-related	0.0210	0.75	
AT3G29090.1	Pectin methylesterase 31 (PME31)	0.0001	0.70	
AT3G16410.1	Nitrile specifier protein 4 (NSP4)	0.0001	−0.80	

**Proteins with expression differentially regulated 30** **min post-treatment**				
AT1G52300.1	Zinc-binding ribosomal protein family protein (RPL37B)	0.0001	1.00	A
AT4G23670.1	Polyketide cyclase/dehydrase and lipid transport superfamily protein	0.0001	0.85	D
AT5G17820.1	Peroxidase superfamily protein (Prx57)	0.0001	0.65	C, E
AT2G37970.1	SOUL heme-binding family protein (SOUL-1)	0.0008	−0.70	
AT2G41730.1	Unknown protein	0.0002	−0.80	

Only genes showing significant (*p*-value <0.05; Mann–Whitney test) differential expression, with at least 1.5-fold change in at least 2 out of 3 biological replicates, are presented. Gene Ontology (GO) terms include biological process (BP) category. A—Translation (GO:0006412). B—Cellular membrane fusion (GO:0006944); C—Oxidation-reduction process (GO:0055114); D—Response to salt stress (GO:0009651); E—Root hair elongation (GO:0048767); AGI—Arabidopsis Genome Initiative.

**Table 2:** Annotation of proteins differentially expressed 10 min and 30 min after treatment of cells with 10 pM AtPNP-A peptide.

**AGI ID**	**Protein annotation**	***p*****-Value**	**Log**_**2**_**fold change**	**GO term (BP)**
**Proteins with expression differentially regulated 10** **min post-treatment**				
AT5G08040.1	Mitochondrial import receptor subunit TOM5 homolog (TOM5)	0.0000	1.40	
AT3G55010.1	Phosphoribosyl-aminoimidazole synthetase (PUR5)	0.0001	0.85	F
AT1G54410.1	Dehydrin family protein (HIRD11)	0.0001	0.80	
AT1G17880.1	Basic transcription factor 3 (BTF3)	0.0000	0.70	D
AT1G09795.1	ATP phosphoribosyl transferase 2 (ATP-PRT2)	0.0000	−0.80	
AT5G14340.1	Myb domain protein 40 (MYB40)	0.0000	−0.83	
AT4G14430.1	Indole-3-butyric acid response 10 (IBR10)	0.0000	−0.93	E, G
AT1G07660.1	Histone superfamily protein	0.0001	−0.93	


**Proteins with expression differentially regulated 30** **min post-treatment**				
AT1G78150.1	Unknown protein	0.0027	0.75	
AT3G49601.1	Unknown protein	0.0071	0.65	
AT3G04184.1	Unknown protein	0.0280	−0.63	
AT5G41520.1	RNA binding Plectin/S10 domain-containing protein	0.0090	−0.64	A
AT4G23895.3	Pleckstrin homology (PH) domain-containing protein	0.0004	−0.70	
AT3G28710.1	ATPase, V0/A0 complex, subunit C/D	0.0041	−0.86	
AT3G62250.1	Ubiquitin 5 (UBQ5)	0.0008	−1.15	A

Only genes showing significant (*p*-value <0.05; Mann–Whitney test) differential expression, with at least 1.5-fold change in at least 2 out of 3 biological replicates, are presented. Gene Ontology (GO) terms include biological process (BP) category. A—Translation (GO:0006412). D—Response to salt stress (GO:0009651); E—Root hair elongation (GO:0048767); F—Nucleotide biosynthetic process (GO:0009165); G—Response to water deprivation (GO:0009414); AGI—Arabidopsis Genome Initiative.

## Supplementary materials

Supplementary data Supplementary Table 1. Proteins differentially expressed upon cell treatment with 1 nM AtPNP-A and the key to 1 nM AtPNP-A treatment spectra. Supplementary Table 2. Proteins differentially expressed upon cell treatment with 10 pM AtPNP-A and the key to 10 pM AtPNP-A treatment spectra.
